# Recognition of Facial Emotion Expressions in Patients with Depressive Disorders: A Functional MRI Study

**DOI:** 10.3390/tomography9020043

**Published:** 2023-02-27

**Authors:** Sergey Ternovoy, Dmitry Ustyuzhanin, Merab Shariya, Alena Beliaevskaia, Ernesto Roldan-Valadez, Rodion Shishorin, Roman Akhapkin, Beatrice Volel

**Affiliations:** 1 National Medical Research Center of Cardiology, 121552 Moscow, Russia; 2 I.M. Sechenov First Moscow State Medical University, 119435 Moscow, Russia; 3 Directorate of Research, Hospital General de Mexico “Dr Eduardo Liceaga”, Mexico City 06720, Mexico; 4 Serbsky National Medical Research Center of Psychiatry and Narcology, 119034 Moscow, Russia

**Keywords:** functional MRI, emotion recognition, depression, antidepressant therapy

## Abstract

Background: The present study evaluated the cortical activation during emotional information recognition. Methods: The study group included 16 patients with depression, and 16 healthy subjects were enrolled as a control group. Patients received eight weeks of antidepressant therapy. Functional MRI evaluated the cortical activation twice in the patient group and once in the control group. The fMRI task processed the emotional information with face demonstration from the PennCNP test battery. Results: During the processing of emotional information, patients showed activation in the middle and the inferior frontal gyri, the fusiform gyrus, and the occipital cortex. After treatment, patients showed a significant decrease in the frontal cortex activation for negative face demonstration and no frontal activation for positive emotion recognition. The left superior temporal gyrus activation zone appeared in patients after treatment and in the control group. Healthy subjects showed more intense frontal cortex activation when processing neutral emotions and less when showing happy and sad faces. Activation zones in the amygdala and the insula and deactivation zones in the posterior cingulate cortex were revealed in the controls. Conclusion: This study confirms the hypothesis that anomalies in the processing of emotional stimuli can be a sign of a depressive disorder.

## 1. Introduction

Impaired emotion recognition and regulation plays a crucial role in the pathogenesis of depressive disorders [1]. The concept of depression as a disorder of affective cognition with a distortion of perception, processing, and synthesis of emotional information is becoming increasingly widespread among researchers [2]. Identifying the mechanisms linking emotional dysregulation and disorders of affective cognition are of great importance not only for revealing the pathogenesis of depression but also for optimising the therapeutic strategy. 

Neuronal models of depression supposed that negative emotions are associated with frontal-limbic connectivity dysfunction [3], manifested in the hypoactivity of the frontal brain regions such as the dorsolateral prefrontal cortex and the dorsal anterior cingulate cortex, and hyperactivity of the ventral/rostral anterior cingulate cortex and the amygdala [4,5].

The recognition of emotional facial expressions is a standard paradigm for studying the features of processing emotional information [6]. Distortion of the processes of recognising facial emotions in depressive patients can lead to social isolation, a sense of interpersonal rejection, and restriction of nonverbal expressiveness [7].

Brain imaging techniques, such as functional magnetic resonance imaging (fMRI), can significantly contribute to understanding how facial expressions are processed in humans. However, the results obtained in individual studies remain contradictory [8]. For example, some studies have found decreased frontal activity in patients with depression compared to the control group [9,10]. Simultaneously, other studies have shown an increase in the activity of the frontal cortex during the processing of negative facial expressions [11]. Conflicting results were obtained regarding the activation of the limbic system in response to negative stimuli. Several studies reported an increase in limbic activity in individuals with depression compared to the control group [10,12], while others did not find similar differences [13,14]. Inconsistencies between the results of studies can occur due to heterogeneity of the patient samples (for example, according to the severity or clinical form of the disease), different paradigms for fMRI, and the use of other neural models to interpret the imaging results.

This study was an fMRI radiology sub-study of a larger multidisciplinary research project that included a clinical psychiatry section, an fMRI section (currently underway) and the following study (currently in progress) to evaluate the effect of transcranial stimulation on the identified cortical zones.

The clinical section of this study showed that patients with depression are slower to identify positive emotions but have a similar time recognising negative emotions as patients without depression [15]. This sub-study aimed to evaluate the brain activation/deactivation patterns using fMRI in patients with depression during emotion recognition and to compare it between depressive patients before and after treatment and controls.

## 2. Materials and Methods

### 2.1. Participants

Participants (*n* = 32; mean age of 37.3 ± 9.7 years) originated from the prospective, observational study of the Recognition of Facial Emotions Expressions in Patients with Depressive Disorders [15]. The study group included 16 patients with single or recurrent depressive episodes without psychotic symptoms who scored at least 15 points on the Montgomery-Asberg Depression Rating Scale (MADRS) [16]. Sixteen subjects without depressive disorders were also recruited as a control group. The absence of depression was confirmed during the interview using the MADRS (less than 7 points). A detailed description of the study design, the exclusion criteria, the recruitment procedure, and the treatment strategy are described in a previous publication [15].

The patients’ recruitment was performed at Serbsky National Medical Research Center of Psychiatry and Narcology, Moscow, Russia. The control group was enrolled at Sechenov University, Moscow, Russia. The fMRI study was conducted at the National Medical Research Center of Cardiology, Moscow, Russia. 

The patient’s demographic details are shown in Table 1.

### 2.2. Ethics Approval

The study protocol was approved by the Serbsky National Medical Research Center of Psychiatry and Narcology Ethics Committee (Ref: 10/2/1999), the Sechenov University Local Ethics Committee (Ref: 01-22/2020), the Independent Ethics Committee for clinical trials from the National Medical Research Center of Cardiology (Ref: 262/2020). All participants provided informed consent according to the Declaration of Helsinki.

### 2.3. Data Acquisition and Analyses

#### 2.3.1. Functional MRI Protocol

Functional MRI scans and the severity of depressive symptoms evaluation using the MADRS were performed twice in the patients’ group at baseline and after eight weeks of antidepressant therapy and once in the controls.

**Figure 1:**
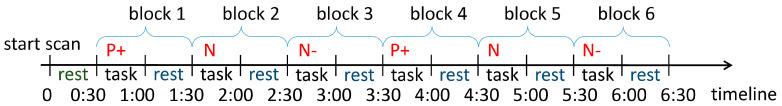
The fMRI paradigm design. The first rest period (indicated in green) was discarded to ensure reaching the T1 equilibrium. Six consecutive blocks were analysed. P+, demonstration of positive, happy faces; N, a task with neutral emotionless faces; N-, a task with negative, sad faces.

MRI data were collected using a 3.0 T MRI scanner Philips Achieva TX with a standard phased-array head coil. Anatomical scans (3D high-resolution T1-weighted pulse sequence with TR = 7.5 ms, TE = 3.5 ms, FOV = 25 × 25 × 15 cm^3^, matrix 228 × 228, flip angle = 8°, slice thickness = 1.1 mm, 250 slices per scan) and functional scans (T2*-weighted gradient-echo EPI pulse sequence with TR = 3000 ms, TE = 35 ms, FOV = 23 × 23 × 12 cm^3^, matrix 96 × 96, flip angle = 90°, slice thickness = 4 mm, 30 slices per scan) were performed [17]. Ten image volumes (10 × 3 s = 30 s) were acquired for each task and rest period (Figure 1). One hundred and thirty image volumes were obtained with a functional scan duration of 6 minutes and 30 seconds.

#### 2.3.2. Emotion Recognition Task

Each trial consisted of six consecutive blocks: an emotion recognition task (30 s) and a rest period (30 s). We used the standardised images of 8 faces expressing happiness, sadness, or no emotion from the Pennsylvania Computerized Neuropsychological Test Battery (PennCNP) that was recommended for use in neurocognitive studies. Examples of images can be found through a link [18]. Each task’s specific emotion was displayed following the paradigm design (Figure 1). 

All participants were instructed and trained to perform the experimental tasks. Face images for the tasks and the commands for rest periods were visually presented using the image projection in the magnet device. The first 30 s rest period was discarded from analysis to ensure the T1 equilibrium.

#### 2.3.3. fMRI Data Processing

Functional MRI data were processed using Statistical Parametric Mapping version 12 (SPM12, http//www.fil.ion.ucl.ac.uk/spm, accessed on 20 February 2023, the Functional Imaging Laboratory, University College, London) running in MATLAB r2014b (the MathWorks, Natick MA, USA). fMRI scans were pre-processed using a standard algorithm consisting of slice-time correction, realignment and re-slicing, co-registration of the fMRI data to the corresponding anatomical scans, unified segmentation, and normalisation of anatomical and functional data to MNI space, and spatial smoothing using an 8 mm full-width half-maximum Gaussian kernel [19]. Functional activation maps for each subject and the resulting group maps were created using SPM12. 

### 2.4. Statistical Analysis

Patients’ activation differences between two evaluation points were assessed using two-tailed paired *t*-tests for dependent samples. A *t*-test for independent samples was used to compare the patient group and the control group. Regional activation was considered significant at a cluster-level threshold (T) ≥ 2.8 with *p* < 0.05 corrected to control the whole-brain family-wise error rate (FWE) [20]. The MADRS points and functional activation correlation were assessed using multiple regression analysis. Demographic characteristics such as age, education, gender and marital status were compared with the Mann–Whitney U test for the non-normally distributed parameters and the chi-squared test for the comparison of proportions.

## 3. Results

### 3.1. Brain Activation Patterns in Depressive Patients before Treatment (Background)

**Figure 2:**
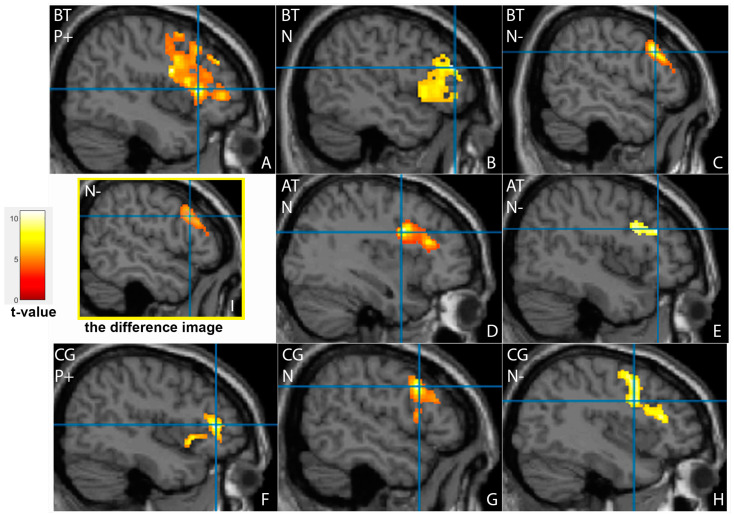
Activation of the frontal cortex in patients before (BT) (**A**–**C**), after (AT) (**D**–**E**) antidepressant therapy and in healthy controls (CG) (**F**–**H**) during facial emotion recognition. Demonstration of positive, happy faces (P+) (**A**,**F**), neutral emotionless faces (N) (**B**,**D**,**G**) and negative, sad faces (N-) (**C**,**E**,**H)**. The corresponding difference image (**I**) showed a significant decrease of activation area in the right middle frontal gyrus for negative faces (*p* < 0.001).

Detailed activation cluster and peak statistics for all study groups can be found in Table 2, Table 3 and Table 4. Before treatment, the participants from the patient group showed significant activation of the frontal cortex (Figure 2A–C). During the processing of emotional information, the activation zones included the left and the right middle frontal gyri, the Brodmann area (BA) 46, and the right inferior frontal gyrus. The frontal cortex, particularly the BA46, is responsible for active thinking. The patients demonstrated the activation of the middle frontal gyri (in the left for neutral faces and in the right for negative emotions) and the right inferior frontal gyrus for neutral and positive emotion processing.

**Figure 3:**
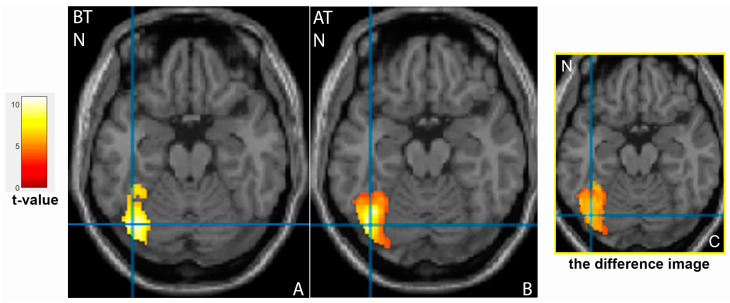
Activation of the left fusiform gyrus (**A**,**B**) in patients before (BT) and after (AT) antidepressant therapy on demonstration of neutral faces (N). The corresponding difference image (**C**) showed a significant decrease of activation area in the left fusiform gyrus, BA37 for neutral faces (*p* < 0.001).

The left fusiform gyrus BA37, considered a face recognition area [21], was activated in patients on demonstration of the neutral faces (Figure 3A). The area of the visual analyser in the occipital cortex was activated in the right inferior occipital gyrus during the processing of negative emotions and in the right occipital cortex (including the primary visual analyser area, BA17) during the processing of positive and negative faces. Additionally, the left middle occipital gyrus and the cuneus activation were revealed to demonstrate happy faces.

### 3.2. Comparison of Brain Activation Patterns in Patients before and after Treatment

After treatment, patients showed a significant decrease of the activation area in the right middle frontal gyrus for the negative faces (extent 386 vs 2064, *p* < 0.001). The right inferior frontal gyrus activation for the neutral faces increased, but the difference was not significant. No frontal cortex activation was revealed for the recognition of positive emotions (Figure 2D–E).

**Figure 4:**
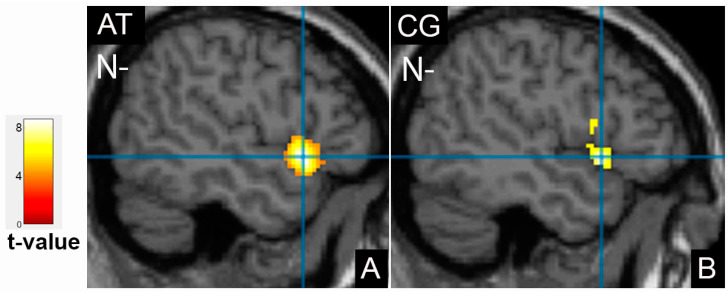
Activation of the left superior temporal gyrus in patients after therapy (AT) (**A**) and in healthy controls (CG) (**B**) on demonstration of negative faces (N-).

Additionally, a significant decrease of the activation area after treatment was found in the left fusiform gyrus, BA37 for the neutral faces (extent 1532 vs 2001, *p* < 0.001, Figure 3B). Additional activation zones were detected in the right fusiform gyrus for the neutral faces and the left superior temporal gyrus for the negative face (Figure 4A).

### 3.3. Comparison of Brain Activation Patterns in Patients before Treatment and Healthy Controls

**Figure 5:**
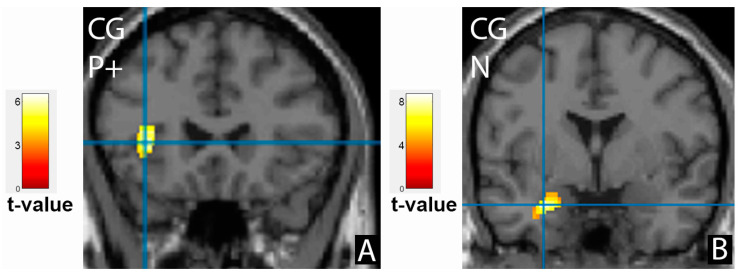
Activation of the left insula (**A**) on demonstration of positive faces (P+) and the left amygdala (**B**) on recognition of neutral emotions (N) in the control group (CG).

**Figure 6:**
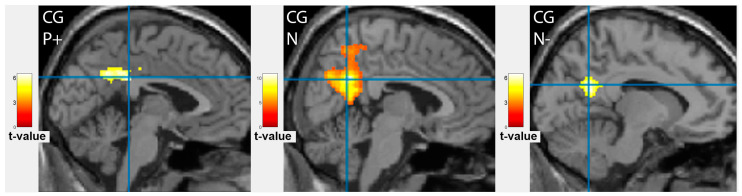
Deactivation zones in the cingulate cortex in the control group (CG), on the left, while processing negative (N-) and positive (P+) emotional information, and on the right, on demonstration of neutral faces (N).

The difference in cortical activation areas in the patient and the control groups did not exceed the statistical significance threshold. However, some promising results should be noted. The healthy subjects showed more intense frontal cortex activation when processing neutral emotions and less when showing happy and sad faces (Figure 2F–H). Activation of the left superior temporal gyrus was observed similarly in patients after treatment, where it was even more evident (Figure 4B). The facial recognition zone in the fusiform gyrus in the healthy volunteers was activated when demonstrating positive emotions (Figure 3C), and the visual analyser in the left middle occipital gyrus showed activation on the neutral faces. In the control group, additional activation zones absent in patients before and after treatment were observed in the left amygdala and the left insula (Figure 5). The deactivation zone of cortical activity was found in the limbic system in the right and left cingulate gyrus (Figure 6).

## 4. Discussion

The main findings of this study are as follows: (a) the activation of the frontal cortex in depressive patients when processing positive and negative emotions decreased after treatment, while healthy controls and patients after treatment showed more intense frontal activation on neutral faces; (b) the activation zones in the insula and amygdala and the deactivation zone in the cingulate gyrus, detected in healthy subjects, did not function in patients both before and after treatment.

More than 25 studies have been published that have evaluated the activation of the cerebral cortex in response to the demonstration of emotional faces in patients with depression. Most of them are investigated in detail in the meta-analysis by Stuhrmann et al. [22]. Although the mechanism of cortical activation in processing emotional information is generally known, there are still unresolved questions in this field. The most important question that remains unclear is whether the detected activation patterns in depressed patients are neurobiological abnormalities or markers of depression that can be corrected during treatment with antidepressants [22]. 

Several studies have reported that the prefrontal cortex is involved in processing emotional information [23,24,25,26,27]. Michael Koenigs et al. considered the prefrontal cortex critical neural substrates underlying depression [28]. Gongying Li et al. observed activation of the left medial frontal gyrus, the right inferior frontal gyrus, the bilateral precentral gyrus, and the bilateral middle frontal gyrus in patients suffering from depression in response to negative emotional stimuli [29]. Simultaneously, several studies have shown activation of the frontal cortex in subjects without depression; this functional zone is regarded as an area responsible for active thinking [30] and working memory [31]. The present study showed activation of the prefrontal cortex in the patient group, which was most intense when demonstrating happy and sad emotions. This phenomenon was not persistent and was significantly compensated due to treatment. In the patients after treatment and in the healthy subjects, activation of the prefrontal cortex was also present, but it was mainly observed when neutral faces were demonstrated. 

The mechanism of the critical role of the prefrontal cortex in depressive states is complex and based on three abnormalities most productively investigated in preclinical models: Anhedonia, negative processing biases, and despair-like behaviour (learned helplessness) [32]. These domains are a product, in part, of executive dysfunction due to dysregulation of the prefrontal cortex. Other studies have showed that depression mediates the association between the insula-frontal functional connectivity and social interaction anxiety [33]. 

The activation zones in the insula and amygdala and the deactivation zone in the cingulate gyrus, which have been demonstrated in previous studies to be involved during the processing of emotional information [25,26,27], were absent in the patient group, unlike the healthy volunteers, and these changes were not corrected as a result of treatment with antidepressants. According to a recent study, the amygdala is associated with photoperiod and seasonal depressive symptoms [34].

The deactivation zones in the limbic cortex in the control group were more intense when recognising neutral emotions but were also present on the demonstration of the happy and the sad faces. The study of M. Spies et al. showed that deactivation of the posterior cingulate cortex and the precuneus in patients with depression is a predictor of an early antidepressant response during the first two weeks [35]. The patients in this current study did not have deactivation of the cingulate gyrus when recognising emotional information, thus the response to the treatment was achieved only after eight weeks. As such, this period was chosen to perform the second point of the fMRI study.

The activation zone in the left superior temporal gyrus was of particular interest. Our study observed this area in the control group and the patients after treatment. Previous studies have revealed that the activation of this area may be a potential biomarker of the antidepressant-related early therapeutic effects underlying the influence of dopamine pathway genes in major depressive disorder [36].

The activation of facial recognition zones in the fusiform gyrus has been described in previous studies [25,27]. Another study demonstrated the functional connectivity of the fusiform gyrus to the frontal and the insular cortices, which was positively correlated with the emotion perception test [37]. A recent resting-state fMRI study confirmed aberrant functional connectivity between the anterior insula and the frontal and the limbic cortices, that was discussed as a possible mechanism underlying somatic depression [38].

The visual cortex activation in the occipital lobes in patients before treatment and the control group could be considered non-specific.

A further unresolved issue concerned the heterogeneity of the presentation paradigms [22]. This study used standardised emotional face images from the Pennsylvania Computerized Neuropsychological Test Battery (PennCNP) [18]. This approach allowed the evaluation of the primary cortical activity patterns, which can be recommended as a standard paradigm for further studies in this field.

### 4.1. Limitations of This Study

Some limitations of this study should be noted. The sample size was small, however it was enough to detect significant cerebral cortex activation in the study groups and compare them at two-time points. The control group included healthy volunteers, and no placebo-controlled group was included. The fMRI study was part of an observational study [15], and it inherited the characteristics of the patient population and treatment strategies. 

### 4.2. Future Directions

In the previous decade, several studies using the morphological features of anatomic MRI images (volumetric asymmetry indices) evinced specific gyri to discriminate between male and female patients [39]. There is also evidence that specific gyri are responsible for the discrimination between the left and right cerebral hemispheres [40]. Some volumetric imaging biomarkers can characterise the group of gyri responsible for episodic memory [41].

Which additional evidence will be needed to consider these fMRI features as contemporary imaging biomarkers for depressive disorders remains a question to be answered. It is logical to propose assessing the correlations between the mentioned gyri with their volumetric parameters and comparing the “difference in correlations” between patients and healthy subjects in a future study. This study will give insight into the latent relations between volumes, asymmetry indices, and fMRI features.

Readers might be interested to know the “diagnostic performance” of the fMRI activation zones discovered and the possible predictive ability of this biomarker to detect or predict depression in healthy subjects. This assessment will approach more of these fMRI biomarkers in day-to-day clinical practice.

Another section of this study is now in progress, in which the results of the transcranial magnetic stimulation (TMS) of the identified cortical zones will be evaluated. A recent review and meta-analysis confirmed evidence of the efficacy of TMS in adolescents with major depressive disorder [42,43].

The overall results of this study may allow us to move from theoretical fMRI studies to a practical approach where the exact information about the cortical activity features in patients with depression will contribute to improving the effectiveness of the treatment of such patients.

## 5. Conclusions

Antidepressant therapy reduced the frontal cortex activation in patients while processing positive and negative emotional information. It increased the frontal cortex activation when demonstrating neutral faces, which corresponded to the results obtained in healthy volunteers. The activation zone in the left superior temporal gyrus observed in patients after treatment also corresponded to the results obtained in the control group. The activation zones in the left insula left amygdala, and the deactivation zone in the cingulate gyrus, identified in the healthy group, were not reproduced in patients after treatment. 

This study confirmed the hypothesis that anomalies in the processing of emotional stimuli can be a sign of a depressive disorder. These data are consistent with previous neuroimaging studies of patients with depression. In this study, we aimed to find an approach to the issues that currently remain unresolved. These data can be considered another step in understanding the pathogenesis of depressive disorders.

## Figures and Tables

**Table 1:** General characteristics of study participants (Akhapkin et al., 2021) [15].

Parameters	Patients (*n* = 16)	Controls (*n* = 16)	*p* Value
Gender, *n* (%)			
Male	2 (12.5)	3 (18.8)	0.87
Female	14 (87.5)	13 (81.3)	
Age, years	37.9 ± 12.5	37.3 ± 11.7	0.64
Education, years	16.3 ± 1.7	16.4 ± 2.7	0.73
Marital status, *n* (%)			
Married	6 (37.5)	13 (81.25)	0.046
Single	9 (56.25)	3 (18.75)	
Divorced	1 (6.25)	0	
Diagnosis of the primary disease, *n* (%)			
Single depressive episode	10 (62.5)	–	N/A
Recurrent depressive episode	6 (37.5)	–	N/A
MADRS mean score at baseline	26.3 ± 4.4	–	N/A

Data are presented as mean ± standard deviation or number and percentage, as appropriate; The Mann–Whitney U test was used for the non-normally distributed parameters, and the chi-squared test was used for comparing proportions; MADRS, Montgomery-Asberg Depression Rating Scale; N/A indicates that none of the patients in the control group had a psychiatric diagnosis, so the statistical criteria were not applicable.

**Table 2:** Cluster and peak statistics for brain activations during facial emotion recognition in patients with depression.

Cluster Location	Emotion	Extent	t-Value	*p* (FWE Corrected)	MNI Coordinates
Patients group before therapy					
Frontal cortex					
Left middle frontal gyrus, BA46	N	1457	6.24	<0.001	−48;38;20
Right inferior frontal gyrus, BA46	N	632	8.83	0.001	50;40;4
Right middle frontal gyrus	N-	2064	10.89	<0.001	54;20;30
Right inferior frontal gyrus	P+	2368	8.91	<0.001	46;22;8
Occipital cortex					
Left fusiform gyrus, BA37	N	2001	10.59	<0.001	−40;−70;−18
Right inferior occipital gyrus	N-	3095	16.45	<0.001	40;−70;−16
Right occipital cortex, BA17	P+	4282	13.27	<0.001	18;−96;−4
Left middle occipital gyrus, Cuneus	P+	2017	10.8	<0.001	−24;−92;6

Emotions: N-, negative; N, neutral; P+, positive. BA, Brodmann area. The *t*-test for independent samples with the family-wise error rate (FWE) correction was used for the analyses [20].

**Table 3:** Cluster and peak statistics for brain activations during facial emotion recognition in patients after antidepressant therapy.

Cluster Location	Emotion	Extent	t-Value	*p* (FWE Corrected)	MNI Coordinates
Patients group after therapy					
Frontal cortex					
Right inferior frontal gyrus	N	1390	10.97	<0.001	38;4;28
Right middle frontal gyrus	N-	386	5.51	0.008	46;30;26
Occipital cortex					
Left fusiform gyrus, BA37	N	1532	6.48	<0.001	−40;−68;−20
Right fusiform gyrus, BA37	N	1861	7.44	<0.001	44;−58;−24
Temporal cortex					
Left superior temporal gyrus, BA22	N-	420	5.94	0.005	−48;12;−4

Emotions: N-, negative; N, neutral. BA, Brodmann area. The *t*-test for independent samples with the family-wise error rate (FWE) correction was used for the analyses [20].

**Table 4:** Cluster and peak statistics for brain activations and deactivations during facial emotion recognition in healthy controls.

Cluster Location	Emotion	Extent	t-Value	*p* (FWE Corrected)	MNI Coordinates
Control group, cortical activation					
Frontal cortex					
Right middle frontal gyrus	N	2228	8.91	<0.001	54;14;34
Right inferior frontal gyrus, precentral gyrus	N-	946	6.05	<0.001	42;6;30
Right inferior frontal gyrus	P+	280	7.29	0.041	46;36;8
Left inferior frontal gyrus, insula	P+	358	5.93	0.014	−36;24;4
Limbic lobe					
Left amygdala	N	298	8.29	0.009	−28;0;−24
Occipital cortex					
Left middle occipital gyrus	N	2977	9.13	<0.001	−40;−78;0
Right fusiform gyrus, BA37	P+	3809	12.41	<0.001	36;−72;−20
Temporal cortex					
Left superior temporal gyrus, BA22	N-	1689	8.4	<0.001	−50;14;−6
Control group, cortical deactivation					
Right limbic lobe, cingulate gyrus, precuneus	N	2851	9.49	<0.001	6;−56;28
Left limbic lobe, cingulate gyrus, BA31, precuneus	N-	159	4.93	0.043	−10;−58;24
Left limbic lobe, cingulate gyrus, BA23	P+	143	4.61	0.072	−2;−36;30

Emotions: N-, negative; N, neutral; P+, positive. BA, Brodmann area. The *t*-test for independent samples with the family-wise error rate (FWE) correction was used for the analyses [20].

## Data Availability

The datasets generated and/or analysed during the current study are available from the corresponding author upon reasonable request.
